# Supplementary material for: Structural basis for specific RNA recognition by the alternative splicing factor RBM5
Source: Nat Commun. 2023 Jul 15;14:4233. doi: 10.1038/s41467-023-39961-w (PMC10349855; doi:10.1038/s41467-023-39961-w)
Supplement: Supplementary file 3 — Reporting Summary [file 41467_2023_39961_MOESM3_ESM.pdf]

nature portfolio

Corresponding author(s): Michael Sattler

Last updated by author(s): Jun 14, 2022

Reporting Summary

Nature Portfolio wishes to improve the reproducibility of the work that we publish. This form provides structure for consistency and transparency in reporting. For further information on Nature Portfolio policies, see our Editorial Policies and the Editorial Policy Checklist.

Statistics

For all statistical analyses, confirm that the following items are present in the figure legend, table legend, main text, or Methods section.

n/a	Confirmed	
☐	☒	The exact sample size (*n*) for each experimental group/condition, given as a discrete number and unit of measurement
☒	☐	A statement on whether measurements were taken from distinct samples or whether the same sample was measured repeatedly
☐	☒	The statistical test(s) used AND whether they are one- or two-sided<br>*Only common tests should be described solely by name; describe more complex techniques in the Methods section.*
☒	☐	A description of all covariates tested
☒	☐	A description of any assumptions or corrections, such as tests of normality and adjustment for multiple comparisons
☐	☒	A full description of the statistical parameters including central tendency (e.g. means) or other basic estimates (e.g. regression coefficient) AND variation (e.g. standard deviation) or associated estimates of uncertainty (e.g. confidence intervals)
☐	☒	For null hypothesis testing, the test statistic (e.g. *F*, *t*, *r*) with confidence intervals, effect sizes, degrees of freedom and *P* value noted<br>*Give P values as exact values whenever suitable.*
☒	☐	For Bayesian analysis, information on the choice of priors and Markov chain Monte Carlo settings
☒	☐	For hierarchical and complex designs, identification of the appropriate level for tests and full reporting of outcomes
☒	☐	Estimates of effect sizes (e.g. Cohen's *d*, Pearson's *r*), indicating how they were calculated

Our web collection on statistics for biologists contains articles on many of the points above.

Software and code

Policy information about availability of computer code

Data collection	Diffraction datasets were collected at beamlines of the European synchrotron research facility (ESRF), Grenoble, France using BsxCuBE beamline software (ESRF). SAXS data for RRM1-ZnF1 (C191G) was also collected at ESRF Grenoble using BsxCuBE.<br>NMR data were collected using Topspin v3.2 (Bruker). SAXS data for RRM1-ZnF1-RRM2 (C191G) apo and in complex with GGCU_12 RNA were collected using Rigaku SAXSLab (v3.0.lr1).
Data analysis	Softwares used: Pymol (version 2.3.1), Chimera (version 1.12), CcpNmr Analysis (version 2.4), GraphPad Prism (version 6), PRIMUS (ATSAS version 3.0.2), CRYSOL (ATSAS version 3.0.2), OmniSEC software, Fiji (Image J, version 1.0), XDS (version March 1, 2015), Phenix (version: 1.15.2), Coot (0.8.9), NMRPipe (Version 1.7), Refmac software from CCP4 software suite (version 7.0), Auto-Rikshaw (version 1.08)

For manuscripts utilizing custom algorithms or software that are central to the research but not yet described in published literature, software must be made available to editors and reviewers. We strongly encourage code deposition in a community repository (e.g. GitHub). See the Nature Portfolio guidelines for submitting code & software for further information.

Data

Policy information about availability of data

All manuscripts must include a data availability statement. This statement should provide the following information, where applicable:

- Accession codes, unique identifiers, or web links for publicly available datasets
- A description of any restrictions on data availability
- For clinical datasets or third party data, please ensure that the statement adheres to our policy

NMR backbone chemical shifts for RRM1 and RRM1-ZnF1S-RRM2 have been deposited to the BMRB under the accession codes 51057 [https://bmrb.io/data_library/summary/index.php?bmrbId=51057] and 51058 [https://bmrb.io/data_library/summary/index.php?bmrbId=51058], respectively. Coordinates and structure factors for the RRM1-ZnF1 apo and RRM1-ZnF1S in complex with GGCU_10 have been deposited in the PDB with accession codes 7PCV [https://www.rcsb.org/structure/7PCV] and 7PDV [https://www.rcsb.org/structure/7PDV], respectively. SAXS data for RRM1-ZnF1S apo, RRM1-ZnF1S-RRM2 apo and RRM1-ZnF1S-RRM2 apo and in complex with GGCU_12 have been deposited to the SASBDB with accession codes SASDM43 [https://www.sasbdb.org/data/SASDM43/], SASDM53 [https://www.sasbdb.org/data/SASDM53/] and SASDM63 [https://www.sasbdb.org/data/SASDM63/], respectively. All source data are provided with this paper in SourceData.xls. The NMR structure of RBM5 RRM2 used for generation of RRM1-ZnF1S-RRM2 + RNA structure model is available in the PDB with accession code 2KLZ [https://www.rcsb.org/structure/2LKZ].

Research involving human participants, their data, or biological material

Policy information about studies with human participants or human data. See also policy information about sex, gender (identity/presentation), and sexual orientation and race, ethnicity and racism.

Reporting on sex and gender	Not applicable. No human participants or human data were involved in this study.
Reporting on race, ethnicity, or other socially relevant groupings	n/a
Population characteristics	n/a
Recruitment	n/a
Ethics oversight	n/a

Note that full information on the approval of the study protocol must also be provided in the manuscript.

Field-specific reporting

Please select the one below that is the best fit for your research. If you are not sure, read the appropriate sections before making your selection.

☒ Life sciences ☐ Behavioural & social sciences ☐ Ecological, evolutionary & environmental sciences

For a reference copy of the document with all sections, see nature.com/documents/nr-reporting-summary-flat.pdf

Life sciences study design

All studies must disclose on these points even when the disclosure is negative.

Sample size	For ITC data, two biological replicates were performed.<br>For ex-vivo splicing assays, three independent biological replicates were initially carried out. Given the close reproducibility and confidence intervals of the results in the initial replicates, we expanded the number of replicates to a minimum of five and verified the consistency of the results obtained, which made us confident that the sample sizes used are sufficient to support the conclusions derived from these data.
Data exclusions	No data were excluded from analysis.
Replication	ITC binding data were performed in duplicates. All attempts at replication were in general successful but some runs were excluded due to a noisy baseline, tiny bubbles causing jumps in baseline or under filling of ITC cell etc. Only in these cases were further replicates necessary.<br>All replicates performed for the ex-vivo splicing assays are included.
Randomization	There was no allocation of groups done in this study and so no Randomization was required.
Blinding	There was no allocation of groups done in this study and so no Randomization was required.

Reporting for specific materials, systems and methods

We require information from authors about some types of materials, experimental systems and methods used in many studies. Here, indicate whether each material, system or method listed is relevant to your study. If you are not sure if a list item applies to your research, read the appropriate section before selecting a response.

Materials & experimental systems

n/a	Involved in the study
☐	☒ Antibodies
☐	☒ Eukaryotic cell lines
☒	☐ Palaeontology and archaeology
☒	☐ Animals and other organisms
☒	☐ Clinical data
☒	☐ Dual use research of concern
☒	☐ Plants

Methods

n/a	Involved in the study
☒	☐ ChIP-seq
☒	☐ Flow cytometry
☒	☐ MRI-based neuroimaging

Antibodies

Antibodies used	Ms mAb to GADPH [6C5] ab8245 from Abcam, T7 tag Antibody HRP conjugate 69048 from Novagen, ECL Antimouse IgG HRP NA931 from Cytiva
Validation	Ms mAb to GADPH [6C5] ab8245 from Abcam: This GAPDH antibody can be used as a loading control antibody. GAPDH is a 146 kDa tetramer composed of four 30-40 kDa subunits. There is no cross-reaction with GAPDH from yeast. Preliminary data indicates that the GAPDH antibody- loading control ab8245 recognizes the monomer (36 kDa) and also the dimer forms of GAPDH, but not the tetrameric form of the protein.<br>Species reactivity: Mouse, Rat, Human<br>Immunogen: Full length native protein (purified) corresponding to GAPDH. Database link: P46406<br>o Positive controls: ICC/IF: HeLa cells, NIH3T3 cells, SV40LT-SMC cells. WB: HeLa, A431, Jurkat, HEK-293, Raji whole cell lysate.<br><br>T7 tag Antibody HRP conjugate 69048 from Novagen: The T7•Tag Monoclonal Antibody is a mouse monoclonal antibody (IgG2b, k) directed against the 11 amino acid (MetAlaSerMetThrGlyGlyGlnGlnMetGly) gene 10 leader peptide expressed by many pET vectors, as well as pSCREEN™ and pRSET vectors. The T7•Tag Antibody HRP Conjugate is prepared by covalent coupling the pure antibody with horseradish peroxidase. The enzyme-conjugated antibody enables detection with only one binding step and eliminates cross-reactivity associated with secondary reagents. The antibody has been qualified for Western blotting and can detect less than 1 ng where the cross-reactivity with bacterial, insect or mammalian cells lysates is negligible.<br><br>ECL Antimouse IgG HRP NA931 from Cytiva: HRP-linked whole Ab (from sheep) Highly species specific HRP-conjugated antibodies optimized for use with Amersham ECL Western Blotting Detection Reagents Highly species-sp - GHC-F.

Eukaryotic cell lines

Policy information about cell lines and Sex and Gender in Research

Cell line source(s)	HeLa CCL-2 ATCC, Hek 293T CRL-3216 ATCC
Authentication	As a biological resource center, ATCC comprehensively performs authentication and quality-control tests on all distribution lots of cell lines.
Mycoplasma contamination	All cell lines were regularly tested for mycoplasma contamination by PCR and tested negative
Commonly misidentified lines<br>(See ICLAC register)	No commonly misidentified cell lines were used in the study.
